# Fragmented sleep relates to hallucinations across perceptual modalities in the general population

**DOI:** 10.1038/s41598-021-87318-4

**Published:** 2021-04-08

**Authors:** Sanne G. Brederoo, Janna N. de Boer, Jacqueline de Vries, Mascha M. J. Linszen, Iris E. C. Sommer

**Affiliations:** 1 Cognitive Neuroscience Center, Department of Biomedical Sciences of Cells and Systems, University Medical Center Groningen, University of Groningen, Internal Zipcode FA32, Antonius Deusinglaan 2, 9713 AW Groningen, The Netherlands; 2 Department of Psychiatry, University Medical Center Groningen, Groningen, The Netherlands; 3 Department of Psychiatry, University Medical Center Utrecht Brain Center, Utrecht University, Utrecht, The Netherlands; 4 Reinier van Arkel Mental Health Institute, ’s Hertogenbosch, The Netherlands

**Keywords:** Comorbidities, Psychology, Sleep, Perception, Psychosis

## Abstract

Although previous studies reported a link between sleep problems and the occurrence of hallucinations, more detailed information is needed to translate this association into clinical practice. This study investigates sleep quality and its relation to prevalence, type, content, and phenomenology of hallucinations, using an online survey in a large population sample (n = 10,299). Based on community-based cluster analysis, four groups could be distinguished that differed in terms of sleep quality. Our results confirm previous studies in showing that poor sleep is associated with the occurrence of hallucinations, and extend previous results on a number of aspects. First, we show that particularly fragmented sleep relates to the occurrence of hallucinations. Second, we show that this is the case for hallucinations across the auditory, visual, olfactory, and tactile domains. Third, our results show that fragmented sleep not only relates to the occurrence, but also to the content, frequency, duration, and associated distress of hallucinations. Finally, compared to poor sleep, good sleep quality is associated with hallucinations that are less negative and disruptive. We conclude that sleep hygiene measures could have a large positive impact on individuals whose fragmented sleep underlies the occurrence of bothersome hallucinations.

## Introduction

Although the occurrence of hallucinations has typically been associated with psychosis, it is now commonly known as a transdiagnostic phenomenon that is also prevalent among non-clinical individuals^1^, with prevalences of non-clinical hallucinations reported to range between 5–15%, depending on age^2^. Clinical and non-clinical hallucinations have been reported to differ, but may share similar underlying mechanisms^3^. Looking at hallucinations in the general population helps to understand such underlying mechanisms, as confounding factors such as medication and hospitalization are largely absent. An important factor that has often been associated with hallucinations is poor sleep^4^.

With regard to hallucinations in the context of psychiatric disorders, a study in patients with schizophrenia showed that disturbed sleep predicted higher severity of next-day auditory hallucinations^5^. In the context of Parkinson’s disease, visual hallucinations tend to co-occur with sleep disturbances such as nightly awakenings and reduced REM-sleep time^6^. In line with this finding, Sinforiani et al.^7^ showed that REM-sleep disorders predisposes individuals with neurodegenerative dementias to hallucinations.

Poor sleep is not only prevalent among people with psychiatric or neurodegenerative disorders^8^, it is also a very common health issue in the general population^9^. Previous studies in non-clinical populations showed a link between poor sleep and the occurrence of hallucinations^10–12^. Sheaves et al.^10,11^ confirmed that insomnia is associated with hallucinatory experiences in a number of non-clinical samples (N = 8525 in 2000; N = 7403 in 2007; N = 1403 in 2016). In addition, Oh et al.^12^ concluded that difficulties falling asleep and early waking were both associated with more than a two-fold increased likelihood of psychotic symptoms (N = 2304).

Given the consistent association between poor sleep and hallucinations, interventions to improve sleep may provide a good addition to therapy of patients suffering from bothersome hallucinations. Alternatively, interventions to improve sleep could be a first step in preventive strategies. To address possible lines of treatment and prevention of hallucinations, detailed knowledge about this association is essential. The aim of the current study is to examine different aspects of sleep and the occurrence of hallucinations across modalities in order to gain a more in-depth understanding of their relation.

Dichotomizing a sample based on a composite sleep score (e.g., ‘insomnia’ versus ‘healthy sleep’) does not do justice to the different types of aberrant sleep patterns that exist. For example, some people may predominantly experience difficulties initiating sleep, while others may suffer from nightly awakenings, and yet others have to deal with decreased sleep duration because they awake very early in the morning^13^. To overcome this, we use a heterogeneous approach to sleep phenotypes^14^. Specifically, we use a data-driven approach to reveal participant groups with different sleep patterns, in which the sleep data themselves are informative on distinguishable clusters or ‘communities’ of participants. As we also appreciate the heterogeneity of hallucinations’ content and phenomenology, we employed the recently developed and validated Questionnaire for Psychotic Experiences (QPE)^15^. This questionnaire also addresses hallucinations in other perceptual modalities than the auditory and visual, and provides information on content, frequency, and experienced distress.

## Results

### Sleep groups

**Figure 1:**
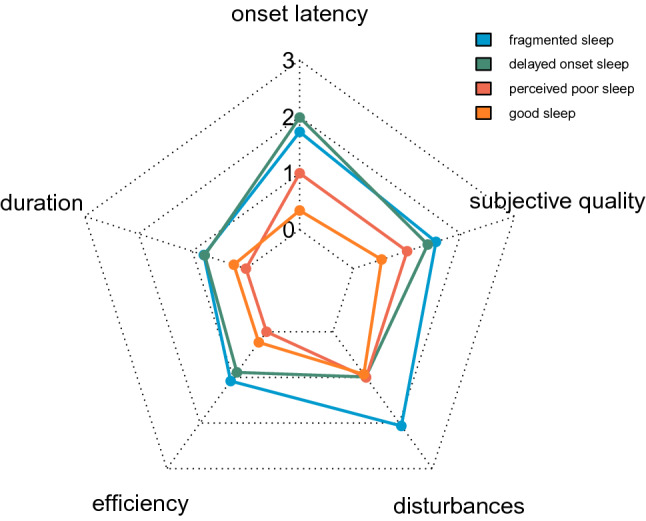
*Sleep characteristics per group.* Averages corresponding to the respective Pittsburgh sleep Quality Inventory domains (ranging from 0 to 3, where 0 is ‘good sleep quality’ and 3 is ‘poor sleep quality’), on which the cluster analysis was based. This figure was created using RStudio version 1.3.959.

The cluster analysis on sleep quality characteristics produced four groups, two of which can be characterized as poor sleepers (the ‘fragmented sleepers’ and the ‘delayed onset sleepers’), and two as good sleepers (the ‘perceived poor sleepers’ and the overall ‘good sleepers’) (Fig. 1).

Model evaluation indicated that the groups could be well distinguished from each other (accuracy κ = 86.7%). Descriptive data of the groups can be found in Table 1. In the following we describe the sleep characteristics and demographic information of these four groups in more detail.

**Table 1:** Demographic information and sleep characteristics.

	Total sample (N = 10,299)	Fragmented sleepers (N = 2867)	Delayed onset sleepers (N = 3220)	Perceived poor sleepers (N = 1257)	Good sleepers (N = 2,946)	*H* / *X*^2^	*p*	Post-hoc analyses*
Sex (% women)	68.9%	80.5%	66.2%	67.2%	61.3%	271	< 0.001	a,b,c,e,f
Age (years) median (IQR)	32 (24)	34 (28)	28 (21)	28 (19)	36 (24)	302	< 0.001	a,b,e,f
Years of education median (IQR)	15 (1)	14 (3)	15 (1)	15 (2)	15 (2)	245	< 0.001	a,b,c,d,e
Daytime function (PSQI) median (IQR)	1 (1)	1 (1)	1 (0)	1 (1)	1 (1)	894	< 0.001	a,b,c,d,e,f
**Sleep**								
Total PSQI score median (IQR)	5 (4)	8 (5)	7 (3)	4 (1)	3 (2)	6430	< 0.001	a,b,c,d,e,f
Duration (hours) median (IQR)	7 (2)	6.75 (1.5)	6.5 (1)	7.5 (1)	7.5 (1)	1755	< 0.001	b,c,d,e,f
Efficiency (PSQI) median (IQR)	0 (1)	1 (2)	1 (1)	0 (0)	0 (0)	2290	< 0.001	a,b,c,d,e,f
Disturbances (PSQI) median (IQR)	1 (1)	2 (0)	1 (0)	1 (0)	1 (0)	9404	< 0.001	a,b,c,d,e,f
Onset latency (minutes) median (IQR)	20 (20)	30 (45)	30 (40)	15 (20)	10 (10)	3301	< 0.001	a,b,c,d,e,f
Subjective quality (PSQI) median (IQR)	1 (1)	1 (1)	1 (1)	1 (0)	0 (1)	3707	< 0.001	a,b,c,d,e,f
Bed time (24 h clock) median (IQR)	23 (1)	23 (1)	23 (1)	23 (1)	23 (1)	47.7	< 0.001	a,c,d,e,f
Get-up time (24 h clock) median (IQR)	7 (1)	7 (1)	7 (1)	7 (1)	7 (1)	22.2	< 0.001	a,c,f
Sleep medication (PSQI) median (IQR)	0 (0)	0 (0)	0 (0)	0 (0)	0 (0)	508	<0 .001	a,b,c,d,e,f

IQR: inter-quartile range; PSQI: score (0–3) from Pittsburg Sleep Quality Inventory; *H* = Kruskal–Wallis test statistic; *X*^2^ = Pearson’s chi-square test statistic; *a: fragmented sleepers differ from delayed onset sleepers; b: fragmented sleepers differ from perceived poor sleepers; c: fragmented sleepers differ from good sleepers; d: delayed onset sleepers differ from perceived poor sleepers; e: delayed onset sleepers differ from good sleepers; f: perceived poor sleepers differ from good sleepers.

#### Fragmented sleepers

Of the four groups, the fragmented sleepers (N = 2,876) are the overall worst sleepers. Participants in this group generally indicate poor sleep quality, but in particular experience more nightly awakenings compared to other participants. The most often occurring nightly disturbances in this group are waking up in the middle of the night or early morning, having to get up to use the toilet, and feeling too hot. Of the participants in the fragmented sleep group, 80.5% are women, which is a higher proportion than in the other groups (all *Χ*^2^ > 84.3, *p* < 0.001). Fragmented sleepers are on average 6 years older than delayed onset sleepers (*U* = 3,819,871, *p* < 0.001) and perceived poor sleepers (*U* = 1,430,802, *p* < 0.001).

#### Delayed onset sleepers

Delayed onset sleepers (N = 3220) can also be considered poor sleepers, but less so than the fragmented sleepers. Compared to the other groups, they score particularly high on sleep onset latency, indicating that they have trouble falling asleep. Of participants in the late sleep group, 66.2% are women. In addition to being younger than fragmented sleepers (see above), delayed onset sleepers are on average 8 years younger than good sleepers (*U* = 3,789,939, *p* < 0.001).

#### Perceived poor sleepers

Participants in this group (N = 1257) cannot be considered poor sleepers (total PSQI < 5), but they evaluate their sleep more negatively than participants in the good sleep group. Compared to the good sleepers, they also indicate to experience more trouble falling asleep, but on the other hand report to sleep longer and have better sleep efficiency. Of the participants in the perceived poor sleep group, 67.2% are women. In addition to being younger than fragmented sleepers (see above), perceived poor sleepers are on average 8 years younger than good sleepers (*U* = 1,415,412, *p* < 0.001).

#### Good sleepers

Participants in this group (N = 2946) are generally good sleepers, and also consider themselves so. Of this group 61.3% are women, which is a smaller proportion than in the other groups (all *Χ*^2^ > 12.8, *p* < 0.001). As reported above, good sleepers are on average 8 years older than delayed onset sleepers and perceived poor sleepers.

### Hallucinations

**Figure 2:**
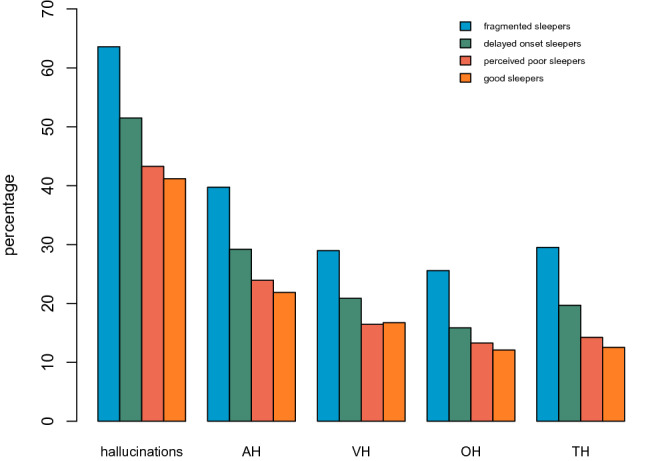
Percentages of participants who experienced at least one hallucination in the past month calculated over all perceptual modalities (‘hallucinations’) and separately for each modality, where AH: auditory hallucinations; VH: visual hallucinations; OH: olfactory hallucinations; TH: tactile hallucinations. This figure was created using RStudio version 1.3.959.

Of the total sample, 50.9% had experienced a hallucination in at least one perceptual modality during the past month (Fig. 2). The proportion of participants who experienced hallucinations during the past month was higher in the fragmented sleepers (63.6%) than in the other groups (all *X*^2^ > 90.3, *p* < 0.001), followed by delayed onset sleepers (51.5%), who differed from the perceived poor sleepers (43.3%) and good sleepers (41.2%) (all *X*^2^ > 24.0, *p* < 0.001). Perceived poor sleepers and good sleepers had the lowest proportions of participants who experienced hallucinations in the past month, and did not differ from each other (*X*^2^_1_ = 1.5, *p* = 0.218). This pattern is similar in each of the four perceptual modalities (auditory [AH], visual [VH], olfactory [OH], tactile [TH]), with fragmented sleepers and delayed onset sleepers differing from the other two groups (all *X*^2^ > 4.4, *p* < 0.034), but perceived poor sleepers and good sleepers not differing from each other (all *X*^2^ < 2.1, *p* > 0.149).

Sleep group membership showed to be a significant predictor for the occurrence of hallucinations in the past month, as evidenced by the logistic regression model (*X*^2^_3_ = 252, *p* < 0.001). The model, adjusted for age, sex, and years of education, explained 10.3% (Nagelkerke’s *R*^2^) of the variance in experienced hallucinations. Results were similar for hallucinations in each of the four perceptual modalities (AH: *X*^2^_3_ = 202, *p* < 0.001, *R*^2^ = 9.1%; VH: *X*^2^_3_ = 127, *p* < 0.001, *R*^2^ = 5.5%; OH: *X*^2^_3_ = 140, *p* < 0.001, *R*^2^ = 5.0%; TH: *X*^2^_3_ = 227, *p* < 0.001, *R*^2^ = 10.6%). Odds-ratios showed that compared to good sleepers, fragmented sleepers were more than twice as likely (OR = 2.2, CI = 2.0–2.5), delayed onset sleepers were about one and a half times more likely (OR = 1.3, CI = 1.2–1.5), and perceived poor sleepers were not more (or less) likely (OR = 0.9, CI = 0.8–1.1) to experience hallucinations. This pattern is similar in each of the four perceptual modalities (Table 2).

**Table 2:** Odds-ratios of experiencing hallucinations compared to good sleepers.

	Fragmented sleepers (N = 2867)	Delayed onset sleepers (N = 3220)	Perceived poor sleepers (N = 1257)
Auditory hallucinations OR (CI)	2.2 (1.9–2.4)	1.3 (1.1–1.4)	1.0 (0.8–1.1)
Visual hallucinations OR (CI)	1.8 (1.6–2.1)	1.2 (1.0–1.3)	0.9 (0.7–1.0)
Olfactory hallucinations OR (CI)	2.2 (1.9–2.5)	1.4 (1.2–1.6)	1.1 (0.9–1.4)
Tactile hallucinations OR (CI)	2.6 (2.2–3.0)	1.4 (1.3–1.7)	1.0 (0.8–1.2)

OR: odds-ratio; CI: 95% confidence interval of the odds-ratio.

In the following, we examine the results in more detail by focusing on those participants who reported to have experienced a hallucination in the past month, per perceptual modality (Table 3).

**Table 3:** Hallucination content and phenomenology.

Auditory hallucinations (% of total)	Total sample	Fragmented sleepers	Delayed onset sleepers	Perceived poor sleepers	Good sleepers	*H*/*X*^2^	*p*	Post-hoc analyses*
	N = 3028 (29.4%)	N = 1143 (39.7%)	N = 940 (29.2%)	N = 301 (24.0%)	N = 644 (21.9%)			
Verbal	62.4%	66.1%	62.3%	55.2%	59.0%	16.7	< 0.001	b,c,d
Single word	33.6%	32.4%	37.3%	33.9%	30.1%	10.2	0.017	a,e
Several words	17.4%	20.7%	15.3%	15.6%	15.5%	13.5	0.004	a,c
Short sentence	13.8%	17.7%	12.1%	12.3%	10.3%	24.0	< 0.001	a,b,c
Complete sentence	5.8%	7.8%	4.4%	3.7%	5.1%	15.0	0.001	a,b,c
Story	6.0%	7.5%	5.2%	5.0%	4.8%	7.9	0.048	a,c
Music	49.4%	54.2%	48.6%	42.9%	45.0%	21.0	< 0.001	a,b,c
Telephone	15.2%	12.4%	16.7%	19.3%	16.2%	12.8	0.005	a,b,c
Duration (QPE) median (IQR)	1 (1)	1 (2)	0 (1)	0 (1)	0 (1)	28.8	< 0.001	a,b,c
Distress (QPE) median (IQR)	0 (1)	1 (2)	0 (1)	0 (1)	0 (1)	208	< 0.001	a,b,c,e,f
Impact (QPE) median (IQR)	0 (0)	0 (0)	0 (0)	0 (0)	0 (0)	111	< 0.001	a,b,c,e
Valence (QPE) median (IQR)	0 (1)	0 (1)	0 (1)	0 (1)	0 (0)	171	< 0.001	a,b,c,e,f
Hypnagogic/hypnopompic	10.6%	11.6%	10.9%	10.3%	8.9%	3.2	0.356	–

Visual hallucinations (% of total)	N = 2205 (21.4%)	N = 833 (29.0%)	N = 672 (20.9%)	N = 207 (16.5%)	N = 493 (16.7%)			
Shadows	44.6%	46.4%	49.2%	47.3%	34.4%	26.1	< 0.001	c,e,f
Human	36.7%	37.5%	34.0%	35.1%	39.7%	4.2	0.239	–
Movement	22.5%	19.3%	24.3%	22.2%	25.6%	9.0	0.030	c
Animal	19.8%	19.3%	17.8%	19.6%	23.4%	5.7	0.128	–
Duration (QPE) median (IQR)	0 (1)	1 (1)	0 (1)	0 (1)	0 (1)	36.1	< 0.001	a,b,c
Distress (QPE) median (IQR)	1 (2)	1 (3)	1 (2)	1 (1)	0 (1)	138	< 0.001	a,b,c,e,f
Impact (QPE) median (IQR)	0 (0)	0 (1)	0 (0)	0 (0)	0 (0)	96.0	< 0.001	a,b,c,e
Valence (QPE) median (IQR)	1 (2)	1 (2)	1 (2)	1 (2)	0 (1)	58.0	< 0.001	a,b,c,e,f
Hypnagogic/hypnopompic	9.1%	11.2%	8.4%	9.2%	7.5%	6.6	0.085	–

Olfactory hallucinations (% of total)	N = 1770 (17.2%)	N = 736 (25.6%)	N = 511 (15.9%)	N = 167 (13.3%)	N = 356 (12.1%)			
Fire	37.1%	38.8%	39.2%	36.4%	30.6%	8.4	0.039	c,e
Food or drinks	14.9%	13.7%	16.3%	14.5%	15.5%	1.8	0.620	–
Perfume	10.8%	11.9%	9.2%	6.0%	12.9%	7.9	0.049	f
Flowers	9.5%	9.5%	8.4%	8.4%	11.5%	2.6	0.457	–

Tactile hallucinations (% of total)	N = 2032 (19.7%)	N = 849 (29.5%)	N = 634 (19.7%)	N = 179 (14.2%)	N = 370 (12.6%)			
Haptic	45.0%	49.2%	42.6%	40.1%	42.0%	10.5	0.015	a,b,c
Formication	24.1%	21.9%	25.9%	26.3%	25.1%	3.9	0.263	–
Tingling	17.2%	13.6%	20.1%	24.0%	17.1%	16.8	< 0.001	a,b
Wind	5.9%	8.1%	5.2%	2.8%	3.2%	16.1	0.001	a,b,c

Data were subset to include only those participants who had experienced a hallucination in the given modality during the past month. Proportion of participants who reported certain hallucinatory content (e.g., verbal AH) is given in percentages. Phenomenology of the hallucinations (e.g., duration) is given in QPE score, where 0 indicates least extreme and 5 indicates most extreme. *H* = Kruskal–Wallis test statistic; *X*^2^ = Pearson’s chi-square test statistic; *a: fragmented sleepers differ from delayed onset sleepers; b: fragmented sleepers differ from perceived poor sleepers; c: fragmented sleepers differ from good sleepers; d: delayed onset sleepers differ from perceived poor sleepers; e: delayed onset sleepers differ from good sleepers; f: perceived poor sleepers differ from good sleepers.

#### Auditory hallucinations

Fragmented sleepers more often heard auditory verbal hallucinations (AVH) in the past month compared to perceived poor and good sleepers (all *X*^2^ > 8.7, *p* < 0.003). Delayed onset sleepers more often heard AVH in the past month than perceived poor sleepers (*X*^2^_1_ = 4.6, *p* = 0.031). Of those hearing AVH, fragmented sleepers more often heard longer utterances than other participants (all *X*^2^ > 4.1, *p* < 0.041). Delayed onset sleepers more often heard single words than fragmented sleepers (*X*^2^_1_ = 5.4, *p* = 0.020) and good sleepers (*X*^2^_1_ = 8.5, *p* = 0.004). Furthermore, fragmented sleepers more often hallucinated music than all other participants (all *X*^2^ > 6.3, *p* < 0.012). In contrast, fragmented sleepers less often hallucinated a ringing telephone than other participants (all *X*^2^ > 4.5, *p* < 0.034).

Furthermore, fragmented sleepers experienced AHs that lasted longer than those of other participants (all *U* > 484,149, *p* < 0.001). Fragmented sleepers also experienced more distress from their AHs (all *U* > 427,469, *p* < 0.001), reported that they had a higher impact on their functioning (all *U* > 482,139, *p* < 0.001), and reported a more negative emotional valence of the AHs (all *U* > 235,929, *p* < 0.006) than other participants. In contrast, good sleepers experienced less distress from their AHs (all *U* > 349,199, *p* < 0.001) and reported a less negative emotional valence (all *U* > 333,459, *p* < 0.001) than other participants. Good sleepers also reported that their AHs had less impact on their functioning than was the case for delayed onset sleepers (*U* = 317,949, *p* < 0.001).

On average, 10.6% of the AHs experienced by the participants were hypnagogic or hypnopompic hallucinations, with no differences between the sleep groups (*X*^2^ = 3.2, *p* = 0.356).

#### Visual hallucinations

Good sleepers less often saw shadows than other participants (all *X*^2^ > 18.9, *p* < 0.005), but more often hallucinated movement than fragmented sleepers (*X*^2^_1_ = 8.6*, p* = 0.014). There were no further differences between the groups with regard to the content of the visual hallucinations they experienced.

Duration of the VHs was longer for fragmented sleepers than for other participants (all *U* > 243,939, *p* < 0.001). Fragmented sleepers also experienced more distress from their VHs (all *U* > 278,859, *p* < 0.001), a higher impact on their functioning (all *U* > 244,679, *p* < 0.001) and reported a more negative emotional valence (all *U* > 254,589, *p* < 0.006) than other participants. In contrast, good sleepers experienced less distress from their VHs (all *U* > 201,119, *p* < 0.001), reported a less negative emotional valence (all *U* > 189,309, *p* < 0.001) than other participants, and reported less impact of the VHs on their functioning than delayed onset sleepers (*U* = 176,590, *p* < 0.001).

An average of 9.1% of the VHs were hypnagogic or hypnopompic hallucinations, with no differences between the sleep groups (*X*^2^ = 6.6, *p* = 0.085).

#### Olfactory hallucinations

Good sleepers less often hallucinated that they smelled fire than fragmented sleepers (*X*^2^_1_ = 8.6, *p* = 0.014) and delayed onset sleepers (*X*^2^_1_ = 8.7, *p* = 0.013). Good sleepers more often hallucinated that they smelled perfume than perceived poor sleepers (*X*^2^_1_ = 5.0, *p* = 0.025).

#### Tactile hallucinations

Fragmented sleepers more often experienced tactile hallucinations resembling touch (all *X*^2^ > 6.3, *p* < 0.042) and the wind (all *X*^2^ > 4.3, *p* < 0.037) than other participants. Fragmented sleepers less often experienced a tingling sensation than delayed onset sleepers (*X*^2^_1_ = 13.3, *p* = 0.010) and perceived poor sleepers (*X*^2^_1_ = 13.7, *p* = 0.009).

## Discussion

Our results indicate that individuals whose sleep can be characterized as fragmented (i.e., frequent nightly awakenings) are more likely to experience hallucinations than individuals whose poor sleep is characterized by other aspects (such as trouble falling asleep) or whose sleep is qualitatively good. Our findings are in line with previous studies that indicated a strong association between psychotic symptoms and sleep problems in the general population^10–12,16^. We extend previous results in showing that this relation holds for hallucinations in the auditory (AH), visual (VH), olfactory (OH), and tactile (TH) perceptual modalities. Moreover, our results show that fragmented sleepers experience more complex and emotionally taxing hallucinations than other individuals, report them to have a larger impact on their functioning and to be more distressed by them. While hallucinations also occur in individuals who sleep well, they seem to be less distressing and impactful than is the case for poor sleepers.

While women were overrepresented in our overall sample (68.9%), this was even more pronounced in the group of fragmented sleepers, and least so in the group of good sleepers. These results are in line with those from a large cross-sectional study (N = 64,503) on the relation between insomnia subtypes and demographic characteristics, which showed that women more often suffer from fragmented sleep than men^13^. The same study, furthermore, indicated that younger individuals who report poor sleep most commonly experience difficulties initiating sleep, a finding that we replicate and relate to higher prevalence of hallucinations.

Due to the resemblance between hallucinations and sleep-related sensations (i.e., dream content, hypnagogic/hypnopompic hallucinations), explanations for their occurrence have been sought in overlapping brain mechanisms. For example, previous observations of the co-occurrence of REM-sleep disturbances and VH in Parkinson’s disease inspired the theory that such hallucinations are ‘REM intrusions’ of visual imagery during wakefulness^17,18^. Our results fail to support this, as fragmented sleepers’ increased odds to experience hallucinations were not limited to the visual domain. Rather, they experienced more hallucinations than other individuals in each of the examined sensory modalities. While auditory sensations have recently been shown to be more common and vivid in dreams than previously thought^19^, the notion that OH and TH could be explained as REM intrusions as well seems unlikely, given that such sensations rarely occur in dreams^20^. As such, our findings are in line with the view that brain mechanisms underlying sleep-related sensations cannot fully account for the daytime occurrence of hallucinations^21^.

With the current study we showed that the relation between poor sleep and hallucinations as found in psychiatry generalizes to the general population. In doing so, we have focused on sleep and hallucinations in isolation from some other possibly relevant aspects. Poor sleep and hallucinations are not standalone factors, but are intertwined with other general mental and physical health aspects (e.g., mood, alcohol and drug intake, personality, and life events), and are both strongly influenced by stress. Together, these aspects can make an individual more or less susceptible to both sleep problems and hallucinations, and determine his or her resilience to their negative effects. Since anxiety and depression may act as mediators of the sleep-psychosis relationship^4^, future studies should address this in the general population.

The fact that the current study is cross-sectional prevents us from drawing conclusions regarding causation. However, previous studies have shown cross-sectional and longitudinal results on the relation between sleep and hallucinations to align^11^, supporting the notion that current sleep quality is predictive of future occurrence of hallucinations. While—due to the cross-sectional nature of our studies—forming no direct evidence, our results do suggest that a first line intervention for individuals who find themselves troubled by hallucinations might be to improve sleep continuity. Such an intervention need not necessarily be effortful; for example, reducing the amount of liquid intake in the evening and regulating room temperature have proven successful strategies in improving sleep^22^. As having to get up to use the bathroom and feeling too hot were the most often reported reasons for fragmented sleep in the current study, such simple sleep hygiene measures may prove effective in light of reducing hallucination occurrence and impact.

This being said, it should be noted that delayed onset sleepers—who experienced relatively few nightly disturbances—also reported more hallucinations than participants who were better sleepers, albeit less so than fragmented sleepers. However, the content of their hallucinations was often less complex (e.g., hearing single words rather than longer utterances), and the hallucinations were less distressing and impactful than was the case for fragmented sleepers. Interventions aimed at reducing rumination, such as meditation^23^ and practicing self-compassion^24^, could help alleviate hallucinations by reducing difficulties falling asleep.

## Limitations

In our study, participants were more often female and highly educated, which could lead to selection bias. Therefore, we adjusted for age, sex and years of education in our analysis. As noted, women on average sleep worse than men, so this could be part of the explanation of the high numbers of poor sleepers in our sample.

It should be noted that we purposely refrained from including the occurrence of nightmares as a variable in our analyses, to prevent circularity, although studies exist that more directly address the relation between nightmares and psychotic symptoms, e.g. Kammerer et al.^25^. The extent of sleep fragmentation is, amongst others, based on the occurrence of nightmares (as one of the questions in the PSQI on which the sleep disturbances subscale is based). Consequently, nightmares more often occur in the fragmented sleepers. Further research is needed to explore the relation between sleep, nightmares and psychotic symptoms in greater detail.

Furthermore, while most of the questions in the QPE were forced choice, some were not. Specifically, questions with regard to the content of OH and TH were open-ended, which are generally considered less advantageous than close-ended questions as they tend to produce more missing data and, logically, result in more diverse answers than forced-choice questions^26^. For this reason, it could be hypothesized that our representation of the content of OH and TH in the general population might be askew. Speaking against this, however, is our corroboration of previous phenomenological assessments of OH in schizophrenia spectrum disorders^27^, by showing a predominance of fire, food-related, perfume, and flower hallucinations. To our knowledge, the phenomenology and content of tactile hallucinations has not yet been thoroughly investigated. However, the presence of small-scale and case studies on formication and haptic hallucinations especially, resonates with our finding that those are the most prevalent types of THs.

## Conclusion

To conclude, our results confirm the strong relationship between sleep problems and hallucinations, and provide further insight into the phenomenological aspects of hallucinations as occurring in the context of poor sleep. Our data suggest that interventions targeted at the improvement of sleep continuity and sleep initiation could be explored as first line interventions for people experiencing bothersome hallucinations. Moreover, not only will improved sleep contribute to a decrease in the frequency of hallucinations, it is also likely to promote resilience against negative effects of hallucinations when they do occur.

## Methods and materials

### Participants

We performed a cross-sectional online survey in The Netherlands between September 2016 and May 2017. Any participant of 14 years or older and able to understand the Dutch language could participate. Participants filled out the questionnaires on a computer or smartphone.

### Ethics statement

The study was conducted in accordance with the Declaration of Helsinki (64th WMA general assembly; October 2013) and the International Conference on Harmonization—Good Clinical Practice (ICH-GCP). The institutional review board (IRB) of the University Medical Center Utrecht approved the study (IRB number: 16–408). Given the low burden of the study, the ethical committee deemed the study not to fall under the Research Involving Human Subjects Act. The Dutch ethical guidelines allow such low-burden epidemiological studies that fall outside the scope of the Research Involving Human Subjects Act to take place without obtaining additional personal informed consent from kin or legal guardians. Therefore, participants of all ages gave written informed consent before participation. Participants were given the option to enter an email-address in case they wanted be contacted for participation in future research. After data collection, any potentially identifying information (i.e., IP-addresses, email-addresses) were removed from the data file and stored separately.

### Survey

A description of the survey is provided elsewhere^28,29^. For the current study, we used two questionnaires; the Questionnaire for Psychotic Experiences (QPE) and the Pittsburgh Sleep Quality Index (PSQI). In addition, we used demographic information regarding age, sex and years of education.

#### QPE

The QPE is a 50-item questionnaire that was designed to quantify psychotic-like experiences, among which hallucinations (i.e., perceptions without a clear source from the environment), and was recently shown to produce valid and reliable results, with good internal consistency (Cronbach’s α ranging between 0.75–0.9 over different subscales)^15^. The QPE investigates hallucinations in the auditory (AH), visual (VH), olfactory (OH), and tactile TH) modalities. In addition to the occurrence of hallucinations, the QPE can be used to investigate content and phenomenology of the hallucinations. Of specific interest for the present study, the QPE asks about the time of occurrence of AH and VH, with ‘around the time of falling asleep or waking up’ being one of the answer options. As such, hypnagogic and hypnopompic hallucinations can be identified using the QPE (albeit not distinguished from each other).

#### PSQI

We used a Dutch version of the PSQI^30^, a validated self-report questionnaire about sleep quality during the past month, with fair internal consistency (Cronbach’s α ranging between 0.7–0.83 across studies)^31^. The PSQI consists of nineteen self-report items, which were used to calculate seven ‘domain’ scores. These domains pertain to five characteristics of sleep quality (i.e., subjective sleep, sleep onset latency, sleep duration, sleep efficiency, and sleep disturbances), and two possible consequences of decreased sleep quality (i.e., use of sleep medication and daytime functioning). Each domain score ranges from 0 (good sleep quality) to 3 (poor sleep quality). The total PSQI score was calculated by adding all scores, thus ranging from 0 to 21, where a total score < 5 indicates overall good sleep quality and > 5 indicates overall poor sleep quality^30^.

### Data

Of the 10,867 valid entries (i.e., excluding duplicates and test entries), 419 were excluded because the participants were younger than 14 years old. Data of another 149 subjects were excluded due to impossible answers on the PSQI (e.g., sleep efficiency of 200%). The analyses were performed on the resulting data from 10,299 participants.

### Statistical analyses

All analyses were run in RStudio version 1.3.959^32^, using the igraph package for the cluster analysis and caret package for model evaluation.

#### Cluster analysis

Community structure detection is a clustering method based in graph theory^33^, that we used to classify the participants into groups based on five characteristics of their sleep quality. These characteristics were sleep duration, sleep onset latency, sleep efficiency, sleep disturbances, and subjective evaluation of sleep, as drawn from the PSQI (i.e., ranging between 0–3). To perform the community structure detection, Euclidean distances were calculated to construct a similarity matrix across the sleep characteristics, representing each participant’s similarity to each other participant. We then used the greedy modularity maximization approach by Newman^34^ to detect which participants grouped together. This approach aims to identify communities in a network by determining which of those share fewer edges in a network than would be expected in a similar network with randomly placed edges.

To assess how well the resulting groups could be distinguished from each other, we employed model evaluation using a support vector machine (SVM) with leave-one-out-cross validation (LOOVC). The resulting accuracy (Cohen’s Kappa) indicates the percentage of participants that were correctly classified into their groups after training the model on the remaining participants, corrected for random chance.

#### Group comparisons

First, we examined how the groups differed in terms of sleep characteristics and demographic information. In large datasets such as these, even small differences often become statistically significant. In our descriptions, we focus only on those differences that can be meaningfully interpreted (e.g., we do not go into differences between groups when their distributions and/or medians are identical or nearly identical). Next, our analyses of interest were those with which we compared the groups in terms of hallucination prevalence, content, and phenomenology. Specifically, we compared the groups in terms of the proportions of participants who had experienced a hallucination in the past month in any perceptual modality, as well as split per modality (AH; VH; OH; TH). Next, per modality and focusing on those participants who had experienced a hallucination in that modality in the past month, we assessed differences between the groups in terms of content of the hallucinations (e.g., verbal or non-verbal AH), and phenomenology of AH and VH (e.g., duration of the hallucination) (see Table 3 for a full list of content and phenomenological aspects). The QPE does not include any questions regarding phenomenology such as duration of OHs and THs, so for hallucinations in these modalities we only investigated group differences in terms of content.

To examine differences between the groups, we used chi-square tests in comparing proportions, and—given the non-normality of the data—Kruskall–Wallis rank sum tests and follow-up Mann Whitney U tests in comparing distributions. Furthermore, we performed binary logistic regression to investigate how membership of a certain sleep group was associated with the occurrence of hallucinations (using odd-ratios), adjusted for sex, age, and years of education. All tests were two-tailed, and alpha was set at 0.05 for all analyses.

## Data Availability

The dataset analyzed during the current study is available on the Open Science Framework, 10.17605/OSF.IO/QDTR7.
